# Disentangle the Causes of the Road Barrier Effect in Small Mammals through Genetic Patterns

**DOI:** 10.1371/journal.pone.0151500

**Published:** 2016-03-15

**Authors:** Fernando Ascensão, Cristina Mata, Juan E. Malo, Pablo Ruiz-Capillas, Catarina Silva, André P. Silva, Margarida Santos-Reis, Carlos Fernandes

**Affiliations:** 1 CE3C – Centre for Ecology, Evolution and Environmental Changes, Faculdade de Ciências, Universidade de Lisboa, Ed C2–5° piso, Lisboa, Portugal; 2 Terrestrial Ecology Group, Departamento de Ecología, Universidad Autónoma de Madrid, Madrid, Spain; 3 Dirección de Innovación y Sostenibilidad, Obrascón Huarte Lain, S.A., Madrid, Spain; Sichuan University, CHINA

## Abstract

Road barrier effect is among the foremost negative impacts of roads on wildlife. Knowledge of the factors responsible for the road barrier effect is crucial to understand and predict species’ responses to roads, and to improve mitigation measures in the context of management and conservation. We built a set of hypothesis aiming to infer the most probable cause of road barrier effect (traffic effect or road surface avoidance), while controlling for the potentially confounding effects road width, traffic volume and road age. The wood mouse *Apodemus sylvaticus* was used as a model species of small and forest-dwelling mammals, which are more likely to be affected by gaps in cover such as those resulting from road construction. We confront genetic patterns from opposite and same roadsides from samples of three highways and used computer simulations to infer migration rates between opposite roadsides. Genetic patterns from 302 samples (ca. 100 per highway) suggest that the highway barrier effect for wood mouse is due to road surface avoidance. However, from the simulations we estimated a migration rate of about 5% between opposite roadsides, indicating that some limited gene flow across highways does occur. To reduce highway impact on population genetic diversity and structure, possible mitigation measures could include retrofitting of culverts and underpasses to increase their attractiveness and facilitate their use by wood mice and other species, and setting aside roadside strips without vegetation removal to facilitate establishment and dispersal of small mammals.

## Introduction

Roads are ubiquitous features of contemporary landscapes worldwide and have a considerable impact on wildlife [1,2]. Roads can cause a barrier effect to animal movement and dispersal by representing a physical obstacle [3], when a large number of animal-vehicle collisions occur [4], or when animals display avoidance behavior towards the road or traffic [5,6]. In all cases, this barrier effect reduces the exchange of individuals between roadside populations, with demographic consequences [7] and possibly causing genetic subdivision [8,9]. This population subdivision, in turn, may accelerate the loss of genetic variation due to random drift and increased inbreeding, potentially leading to local extinctions [10–12].

Assessing the main drivers that influence road avoidance behavior is necessary to better understand and manage this barrier effect [5,13,14]. In particular, it is important to have a clear idea about the role of traffic volume in shaping road avoidance. If higher traffic volumes imply greater avoidance, these roads should be prioritized for mitigation. Research on movement data suggests that the presence of the road itself is the key driver for small mammal avoidance [5], whereas for larger mammals such as the wild boar *Sus scrofa* it is high traffic volume [15]. However, an attempt to disentangle the relative importance of road surface and traffic volume to avoidance behavior has yet to be accomplished. Here, we develop a framework using genetic data to infer the main driver underpinning road avoidance behavior.

**Fig 1:**
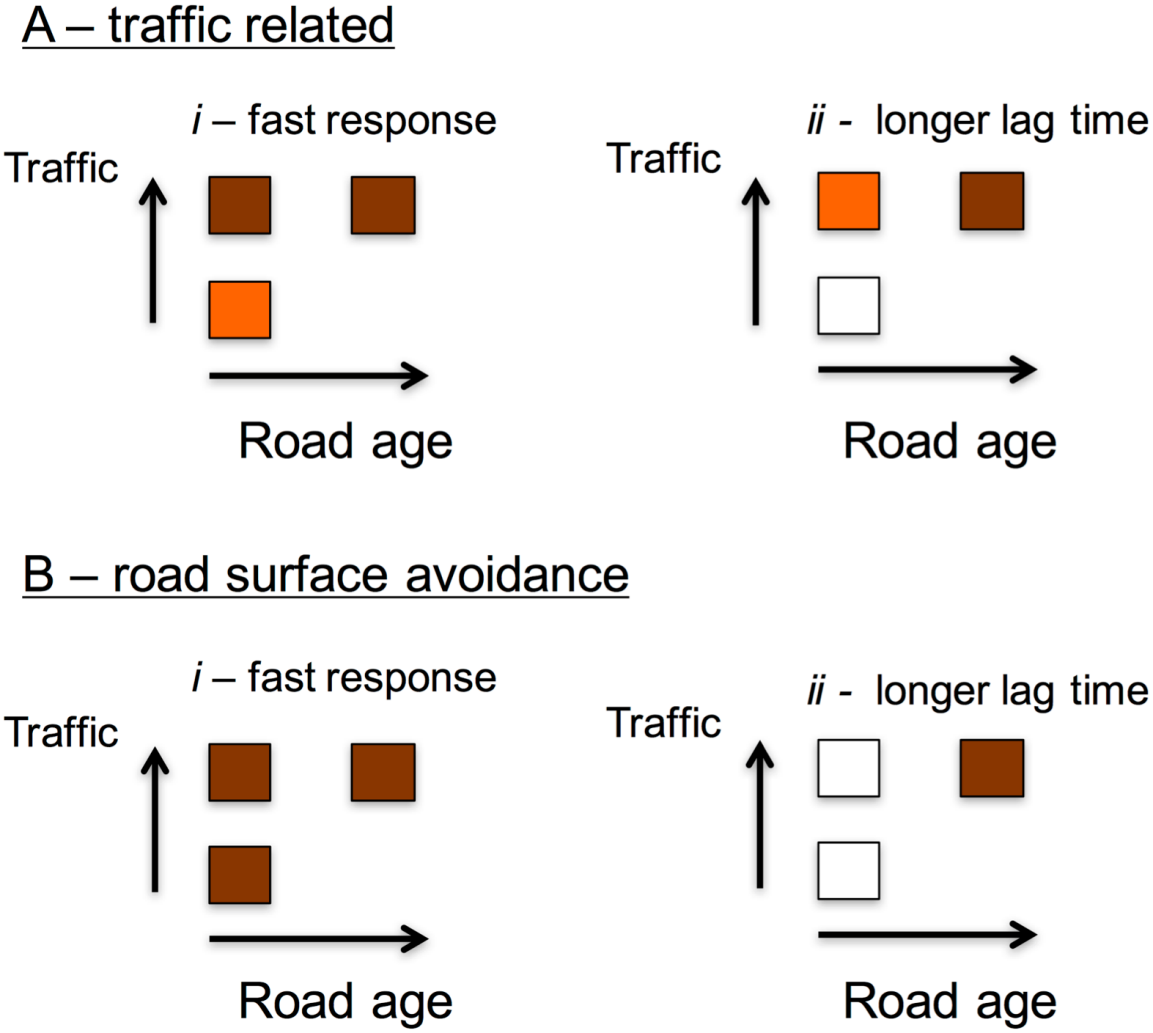
Conceptual representation of the hypotheses tested to explain genetic patterns associated with the road barrier effect. Each square represents a road pictured according to its age and traffic. Darker/lighter color indicates higher/lower genetic differentiation, respectively. (A) Genetic patterns as a response to traffic volume (either due to road mortality or traffic avoidance). (B) Genetic patterns driven by road surface avoidance. In both cases, genetic effects caused by the roads may have short (*i*) or long (*ii*) lag times.

Comparison of genetic patterns has been used to assess population subdivision due to roads [8,9]. Our study is novel in that we implement a sampling design covering similar roads but with different traffic volumes and ages. We hypothesized that if populations on opposite roadsides were genetically differentiated, then lower divergence for roads with lower traffic volumes would indicate that traffic-related factors are the main component of the road barrier effect. However, depending on the temporal lag of the genetic effects, roads with higher traffic volumes but of different ages may exhibit comparable (Fig 1A_*i*_) or distinct (Fig 1A_*ii*_) levels of differentiation. Conversely, if road surface avoidance is the main driver of genetic differentiation, two other scenarios are possible: 1) if genetic responses to road presence are fast, all roads should have similar genetic patterns (Fig 1B_*i*_); or 2) if the genetic effects caused by roads have longer lag times, the roadside populations of older roads should have distinctive (higher) differentiation (Fig 1B_*ii*_).

We focused our hypothesis testing on a small mammal, as this group can be particularly affected by the road barrier effect. For example, several studies using capture-mark-recapture and translocation techniques [5,6,16,17] and movement tracking [18–20] have suggested low crossing rates and/or inhibition of crossing movements. However, to date, few studies have examined the genetic patterns associated with the road barrier effect in small mammals [21–23], and none have evaluated the potential contribution of road avoidance. We were further interested in studying forest-dwelling species as these are more likely to be affected by gaps in cover such as those resulting from road construction. Accordingly, we chose the wood mouse *Apodemus sylvaticus*, a vagile species but for which there is evidence of low road crossing rates [6], as a model for our research. In the Iberian Peninsula the species is common and widespread [24], being especially abundant in evergreen woodlands and shrublands, where it is ecologically important as a common prey item for many predators [25], as a seed consumer [26], and as an acorn disperser [27].

We used microsatellite data to compare genetic patterns among wood mouse populations on the same and opposite sides of three highways of similar width but different traffic volumes and age. To quantify the strength of the genetic patterns, we examined if individuals from opposite highway verges were more genetically distinct than those sampled at a distance several times greater on the same side of the highway. We also estimated probable migration rates between opposite roadside populations. Studies such as this provide valuable insight for the development of appropriate measures to mitigate the road barrier effect, including prioritizing and defining the best locations and designs for wildlife passages, since their effectiveness depends on whether the animals avoid the roads themselves or the traffic they carry.

## Materials and Methods

### Study sites and design

**Fig 2:**
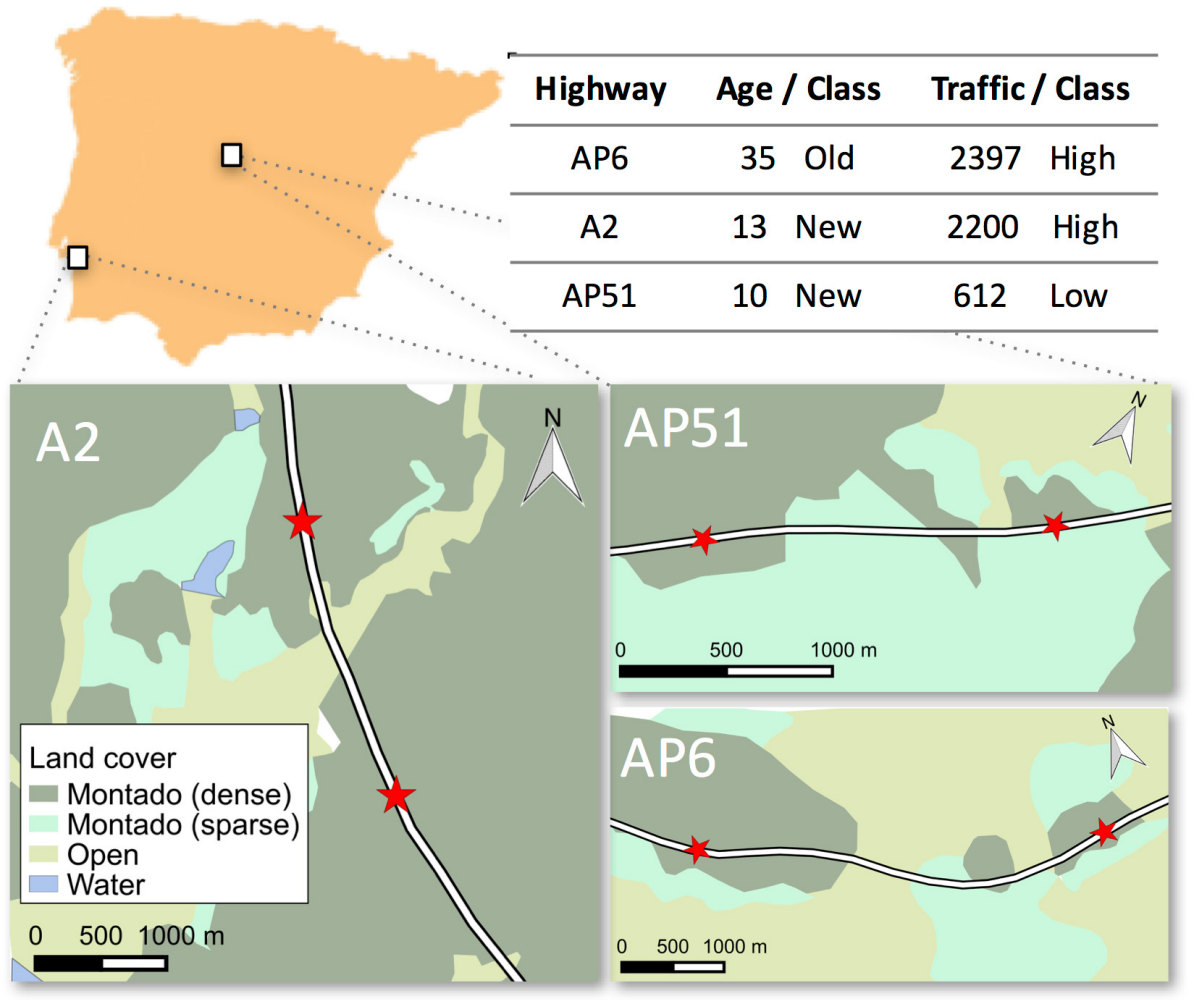
Location of the studied highways (white rectangles) in the Iberian Peninsula, land cover surrounding the study sites (red stars), and classification of the highways according to their age and traffic volume. Land cover was classified based on aerial photographs from Google Maps (accessed on December 1, 2015). Dense/sparse montado (oak woodlands) distinguishes between ‘high’ (>30%) and ‘low’ (<30%) tree cover (authors’ observation). Traffic volume is the number of vehicles passing between 2000 and 0600 h, as the wood mouse is a crepuscular species.

The sampling sites were located along three highways on the Iberian Peninsula, one in Portugal and two in Spain (Fig 2). The width of the right-of-way of the highways was similar (ca. 60 m), including a central median strip (ca. 4 m) and grassy verges (ca. 10 m along each roadside). One highway (AP6) was considerably older and had a high traffic volume, while the other two were of similar age but with differing traffic volumes (Fig 2). Using this sampling design we could control for the three main confounding effects: *(i)* road width, since larger or extra traffic lanes may influence road crossing [16,28], and it has been shown to inhibit crossing movements of small mammals [16,17]; *(ii)* traffic volume, as it may reduce the probability of successful road crossing [28,29] and elicit different responses depending on the species [30]; and *(iii)* road age, as there is a time lag between landscape change and its effect on spatial genetic patterns [31].

We sampled fenced highway verges, which are known to host high densities of wood mice [32,33]. The latter study, for example, estimated an average density over a two-year period near the highway of 5.58 ± 0.75 individuals/ha, against 1.74 ± 0.49 individuals/ha at 500 m from the highway and 3.96 ± 0.99 individuals/ha at 1000 m from the highway. The sampling sites were located in areas where the highways bisected oak woodlands (‘montado’), without any obvious signs of other human disturbance (e.g. urban proximity). The ‘montado’ is a traditional Mediterranean agro-silvo-pastoral system [34], mainly located in the southern regions of Portugal and Spain (where it is called ‘dehesa’). It is characterized by a scattered tree cover of varying density dominated by cork oak (*Quercus suber*) and holm oak (*Q*. *ilex*), with an understory composed of a mosaic of crops, grasslands, shrublands, fallow lands, and by extensive farming and livestock [35]. Shrublands are dominated by species of *Cistus*, *Erica*, *Lavandula*, and *Ulex*. The sampling sites were chosen to be in areas where the highways pass through dense montado (>30% tree cover; authors’ observation), since wood mice may be less abundant in more open woodland [36,37].

For each highway, we analyzed two sampling sites to assess the consistency of genetic patterns, with each site comprising two trap lines (one in each side of the highway). Distance between sites was ca. 3500 m for AP6, 1500 m for A2 and 1200 m for AP51, and the minimum distance between the Spanish highways (AP6 and AP51) was 13 km. Distances between sampling sites were greater than four home range diameters (ca. 1000 m; assuming a circular shape) of the largest territories reported for wood mice, ca. 5.04 ha [38]. Considering that a mean home range area of 1.12 ha has been estimated in oak woodland in the Iberian Peninsula [36], we assumed that the probability of individuals moving between sampling sites was negligible.

Highways in the Iberian Peninsula have numerous regularly spaced culverts (about one per km) for water drainage. Close to the sampling sites, there was a culvert in the first site of A2 and AP6. The first A2 site also had an overpass nearby, while both the first AP6 site and the first AP51 site had a nearby underpass. There was a viaduct in between the A2 sites (ca. 500 m in length and about 100 m from the first site) and in between the AP6 sites (ca. 200 m in length and about 150 m from the second site), and an overpass between the AP51 sites at about 130 m from the first site.

### Animal trapping

Sampling was conducted between February and May of 2010. At each study site, sampling was carried out until about 25 adult wood mice were sampled from each side of the highway, which was generally achieved in less than 15 consecutive sampling days. Each trap line had 60–100 baited Sherman^™^ live traps, placed 10–20 m apart, mostly in the highway verges and where higher capture rates were expected (near oak trees or shrubs), along ca. 400 m of the highway and up to 50 m from the pavement. Where required, oral permission to access private land to conduct the study was obtained from the owners. Permission to access highway verges was obtained from highway companies. Bread fried in rancid oil was used as bait and cotton-nesting material was provided inside each trap as bedding to protect captives from low temperatures. In addition, traps were hidden under vegetation cover to protect animals from adverse weather conditions and to avoid detection by predators. Traps were reviewed at dawn to minimize the time that animals were kept inside.

Each individual captured was weighted, sexed and marked with a fur-clipping code denoting trap line identification, and released at capture location. Individual marking aimed to detect road crossings and to avoid resampling individuals. Individuals of less than 15.5 g were classified as juveniles and those above this weight as adults [39,40]. For each adult we collected approximately 1 cm of the tip of the tail or an ear biopsy, which were preserved in a salt-saturated 20% dimethyl sulphoxide (DMSO) solution [41] for DNA extraction. The cuts were disinfected with antiseptic solution. We did not carry out a specific post-release monitoring of the animals, but we noticed that some first captures already lacked the tip of the tail, possibly due to intra- and interspecific fights or predation attempts. Moreover, 11% of the individuals were recaptured at least once on subsequent days of sampling (see Results) and all seemed to be in good condition. These observations suggest that clipping a small section from the end of the tail should not affect a wood mouse’s survival.

Capture and handling of animals were in conformity with Portuguese and Spanish nature conservation directives (Instituto da Conservação da Natureza e das Florestas in Portugal; Dirección General de Medio Natural in Spain) and in compliance with the European Communities Council Directive 86/609/EEC for animal experiments, and were carried out under the permit of Dirección General del Medio Ambiente de la Junta de Castilla y León (CML/mjg; File: EP/CYL/424/2009) and of Instituto da Conservação da Natureza e das Florestas (254/2009/CAPT). The ethics committee for Animal Care and Use from the Faculdade de Ciências da Universidade de Lisboa—Organismo Responsável pelo Bem-Estar dos Animais, ORBEA—approved all procedures for capture and handling (form 1/2015 ORBEA).

### Microsatellite genotyping

Genomic DNA was extracted from all samples using the EZNA Tissue DNA Kit (Omega BioTek) following the standard protocol for animal tissue. We obtained complete multilocus genotypes by PCR amplification of nine microsatellite loci isolated from *Apodemus sylvaticus*, *A*. *agrarius*, *A*. *flavicollis* and *A*. *draco*: As7, As11, As20 and As34 [42], TNF(CA) [43], SFM2 [44], and SCFM2, SCFM6 and SCFM9 [45] (S1 File). PCR products were run with a GS-500 ROX size standard on an ABI 310 Genetic Analyzer (Applied Biosystems), and scored using Genemapper version 3.7 (Applied Biosystems). Alleles were assigned using bins created in TANDEM version 1.08 [46]. Genotyping was validated by re-amplification and re-analysis of 15% of the samples.

### Data analysis

#### Microsatellite genetic variation

For each sampling line, we checked the microsatellite loci for deviations from Hardy–Weinberg equilibrium (HWE) and linkage equilibrium using, respectively, a probability test and a likelihood ratio test in the program GENEPOP 4.2 [47]. We used α = 0.05 and p-values were adjusted by sequential Bonferroni correction for multiple tests [48]. We used MICRO-CHECKER 2.2.3 [49] to check for allelic dropouts and mis-scoring due to stuttering, and ML-NullFreq to estimate the frequency of putative null alleles using a maximum likelihood method [50]. We calculated number of alleles, observed and expected heterozygosity, and inbreeding coefficients using GENETIX 4.05 [51]. We used GENALEX 6.5 [52,53] to determine the number of private alleles for each sampling line relative to the one on the opposite roadside, with a minimum allele frequency of 0.05 to account for sampling and genotyping errors. Because relatedness may bias estimates of genetic differentiation among populations, we calculated the average pairwise relatedness for each sampling line using the Queller and Goodnight mean estimator [54] in GENALEX. The 95% confidence intervals around the mean values were estimated using 10,000 bootstraps.

We evaluated the statistical power of the microsatellite data set to detect population differentiation using POWSIM 4.1 [55]. This program simulates genetic sampling from populations that have drifted apart to a particular expected value of *F*_ST_, specified by the user, and the population samples are used for testing the null hypothesis of genetic homogeneity. We tested three scenarios for an *F*_ST_ of 0.008 (the lowest observed value; see Results), with allele frequencies and sample sizes as for each of the sampling sites with the lowest *F*_ST_ between opposite roadsides. One thousand simulated data sets were generated for each scenario, and the proportion of significant outcomes (P<0.05), i.e. an estimate of power, was determined using Fisher’s method to combine exact P-values across loci.

#### Comparing genetic signatures of the road barrier effect

To compare genetic differentiation patterns between highways we performed different analyses, as the lag time for detecting a new barrier can be highly dependent on the analytical techniques used [31,56,57]. These analyses focused on three classes of methods: exact tests of population differentiation, *F*_ST_ and related measures, and allele frequency-based genetic distance.

Genetic differentiation was tested in GENEPOP using a log-likelihood (G)-based test [58], without assuming random mating within samples. We used Fisher’s method and a generalized binomial test implemented in MultiTest 1.2 [59] to evaluate overall significance across loci.

We estimated *F*_ST_ [60] and, because this estimator of genetic differentiation can be influenced by the amount of within-population variation, also *G”*_ST_ [61] and *D*_EST_ [62] using GenoDive 2.0b24 [63]. Significance was tested using 20,000 permutations, and 95% confidence intervals (CIs) were obtained by bootstrapping across loci. We used FreeNA [64] to obtain genetic differentiation (*F*_ST_) estimates with an ENA (excluding null alleles) correction. We also used the chord distance (*D*_c_) [65] to measure genetic differentiation. *D*_c_ has been applied in landscape genetic studies [21,66], being robust to low null allele frequencies [64]. Also, allele frequency-based genetic distances may be more sensitive than *F*_ST_ in detecting the effects of recent barriers to gene flow [31]. Chord distances were calculated with FreeNA and 95% CIs were obtained by bootstrapping over loci (1000 replicates).

For the genetic differentiation estimates, we compared results across roadsides against those from same roadside using a differentiation index (dGD) [67,68]. We computed dGD as the mean differentiation between trap lines on opposite sides of the highway minus the mean differentiation between trap lines on the same side of the highway. Positive values of dGD indicate that individuals from opposite roadside trap lines are more genetically distinct than those from same-roadside trap lines. However, as sampling sites were not equidistant we could not control for distance effect and therefore a negative value was not informative. We then bootstrapped individuals 1000 times for significance levels. Under the null hypothesis of no differences between opposite and same-roadside trap lines, dGD should follow a normal distribution centered on zero. Conversely, under the alternative hypothesis (i.e. higher genetic differences among individuals from one of the groups), dGD should be significantly different from zero. We considered the difference to be significant when more than 95% of the test values were greater or less than zero. To test independence of the results from the chosen metric, dGD was performed using both Dc and FST.

#### Estimating migration rates between opposite roadsides

To estimate migration rates between opposite roadsides, we carried out computer simulations parameterized for five variables (Table 1). Simulations consisted of two populations initially exchanging 50% of individuals in each generation (‘pre-highway’) and then we introduced a linear barrier (‘highway’) that bisected the landscape, after which the two populations (roadsides) exchanged 20%, 10%, 5%, 1% or 0% of individuals.

**Table 1:** Simulation parameters, default values and range used in sensitivity analysis.

Parameter	Description	Default values	Variation range
Population size	Number of individuals, half males and half females, in each population	54	20–300
Initial alleles	Initial number of allelic states in each population	30	5–35
Mutation model	Microsatellite mutation model	SMM	SMM, TPM, KAM
Mutation rate	Mutation rate of microsatellites	0.0005	0.00005–0.005
Same-roadside immigrants	In each generation, *n* immigrants from unsampled populations, with allele frequencies identical to the initial ones of the simulated populations, are added to each population (replacing the same number of randomly selected residents)	1	1–5

SMM—stepwise mutation model [69];

TPM—Two-phase model [70];

KAM—*K*-allele model [71].

Simulated populations had 54 individuals (mean value across sampling lines of effective population size, *N*_e_, estimates from LDNe [72], see Table A in S2 File), characterized for nine microsatellite loci, with maximal initial variability and a maximum of 30 possible allelic states (‘Initial alleles’ in Table 1). We assumed free recombination between loci, a stepwise mutation model [69], and a mutation rate of 0.0005 [73,74]. For simplicity, we assumed discrete generations, a single reproduction per lifetime, equal sex ratio, random mating and constant size in each population. We also considered that road mortality could be discounted given the evidence of low road crossing rates in wood mice (see Results and [6,39,40]). Since it is unlikely that roadside populations are completely isolated from adjacent (unsampled) populations on the same side of the highway [23,75], a number of same-roadside immigrants replaced a same number of randomly selected residents in each generation (see Table 1 for details).

Simulations were run for 1000 generations and the barrier was introduced at generation 500. One thousand replicates were computed for each of the five tested between-roadside migration rate values. Simulations were performed in a model written in NetLogo 5.3 [76] that mimics EASYPOP [77] capabilities and options, but allows more flexibility in parameter settings and output (code available in S3 File). For each migration rate scenario, we calculated the values of *F*_ST_, *G”*_ST_, *D*_EST_ and observed heterozygosity (*H*_o_) in each generation, based on samples of 25 individuals from each population. From these datasets we plotted the 5–95% envelope and superimposed the observed values for comparison.

While the population sizes were set based on the estimates of *N*_e_ for the roadside sites, for the other parameters we did not have empirical results and thus the chosen values were taken from the literature (mutation rate) or subjective guesses (e.g. same roadside immigrants). Therefore, in order to understand how the model parameters could have affected our results, we performed a sensitivity analysis to explore multiple model parameterizations (within a range of alternatives to the default parameter values, Table 1). In the sensitivity analyses the parameter under test had a random value within the respective variation range, while the other parameters were set to default values. We ran 500 simulations for each combination of parameter and migration rate (25 combinations in total).

## Results

### Animal trapping

We captured 386 wood mice, 6% of which were classified as juveniles. Forty-four individuals were recaptured at least once (total number of recaptures = 226), but there were no recaptures on opposite sides of the highways, i.e. no road crossings were detected.

### Microsatellite genetic variation

We genotyped 302 adults (n = 101 in AP6, n = 101 in A2, n = 100 in AP51), corresponding to a minimum of 23 samples per sampling line. There was no missing data (S1 File). The error rates per allele and per genotype [78,79,80] were 0.016 and 0.032, respectively. Forty-three percent of the genotyping error was associated with locus As11. Deviations from HWE were observed in zero to three loci across sampling lines, being more frequent at locus As11 (Table B in S2 File). Results from MICRO-CHECKER and ML-NullFreq indicated a higher frequency of null alleles at the same locus, but the estimated null allele frequencies for As11 were greater than 0.1 only in four of the 12 sampling lines, and never exceeded 0.2 (Table C in S2 File). Moderate null allele frequencies (< 0.2) should have a limited effect on measures of genetic diversity and differentiation over all loci [81,82]. The fact that corrected *F*_ST_ values using FreeNA were nearly identical to the uncorrected values (see below) also suggested that the possible presence of null alleles did not introduce a significant bias in our analyses. Significant linkage disequilibrium was found only between loci As20 and As34 at two sampling lines of highway A2 and between As20 and SCFM9 at two sampling lines of highway AP6. Therefore, we decided not to exclude any loci from further analyses. Overall, our data indicated similar levels of genetic variation, inbreeding and relatedness among the 12 sampling lines, with the mean number of alleles per locus ranging from 13.3 to 17.0, the mean observed heterozygosity from 0.76 to 0.85, the inbreeding coefficient from 0.09 to 0.17, and the mean pairwise relatedness from 0.02 to 0.06 (Table D in S2 File). These results suggest that inbreeding and relatedness within sampling lines did not contribute substantially to the observed patterns of genetic differentiation. Measures of microsatellite diversity were comparable to those found in other population samples of wood mice [42,83,84]. The number of private alleles varied between trap lines, but differences were not statistically significant (χ^2^ test = 12.6, df = 11, p > 0.32). Based on the POWSIM analyses, the statistical power of our microsatellite panel for detecting genetic differentiation was high, with a probability of 85% or more of detecting *F*_ST_ ≥ 0.008.

### Comparing genetic signatures of the road barrier effect

**Fig 3:**
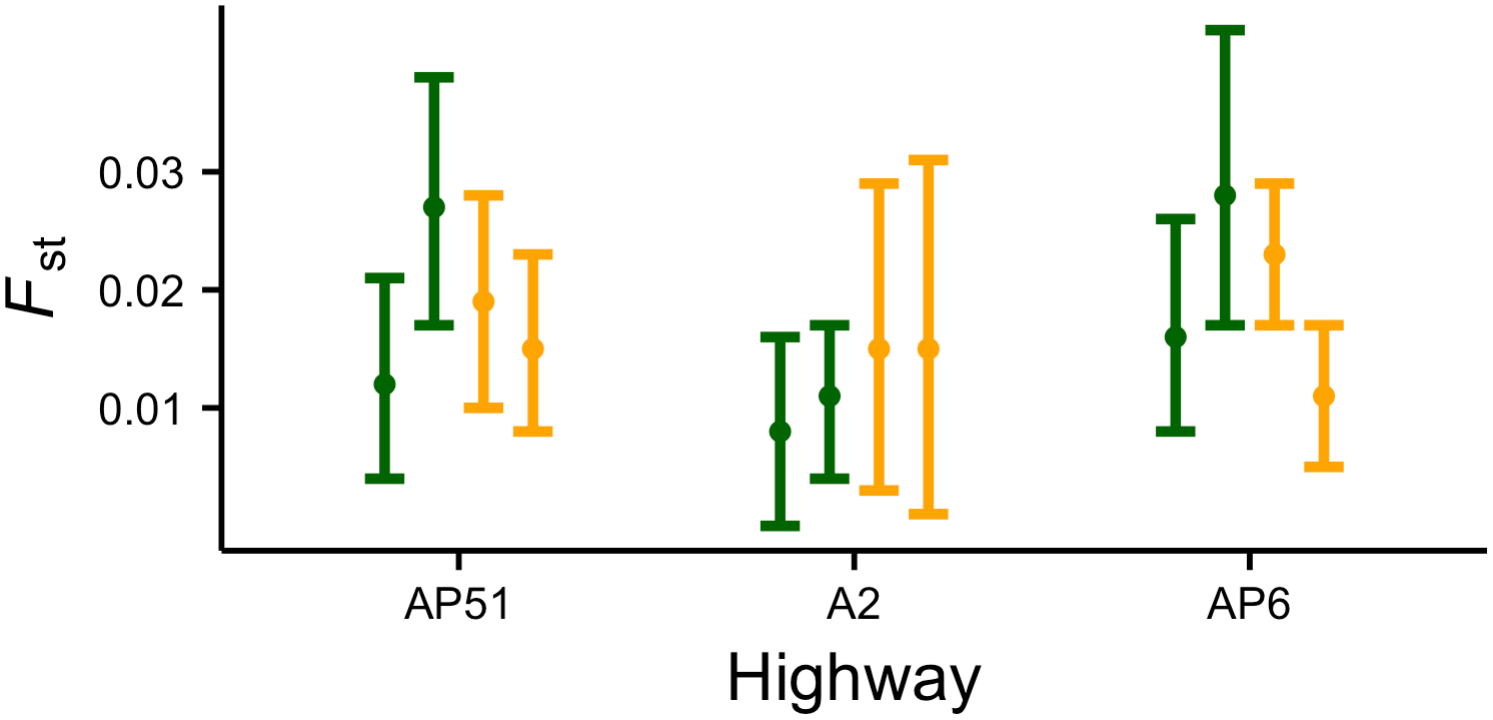
Comparison of genetic patterns for the three highways obtained from *F*_ST_. Green and orange lines represent the genetic differentiation observed between, respectively, sampling lines on opposite roadsides and on the same roadside. Dots represent mean values and limits are 95% confidence intervals.

**Fig 4:**
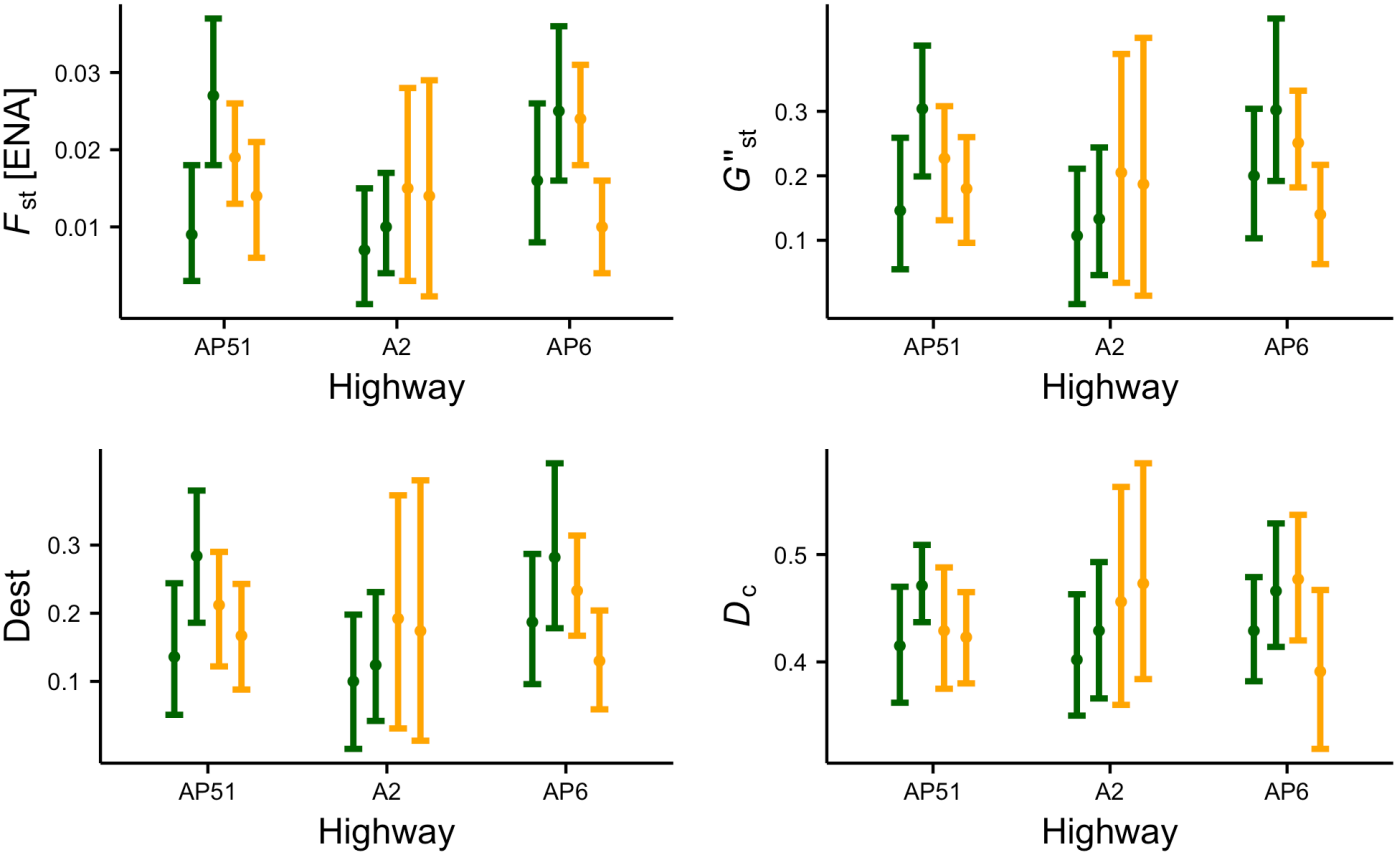
Comparison of genetic patterns for the three highways obtained from *F*_ST_ using ENA, *G”*_ST_, *D*_EST_ and *D*_c_. Green and orange lines represent the genetic differentiation observed between, respectively, sampling lines on opposite roadsides and on the same roadside. Dots represent mean values and limits are 95% confidence intervals.

The G-tests indicated genetic differentiation (p<0.05 in all cases) both between opposite roadsides and same-roadside sampling lines for all three highways. This was corroborated by the significant values of *F*_ST_ and related metrics (p<0.05) and the estimates of genetic distance *D*_c_ (Figs 3 and 4; Tables E and F in S2 File). Values of *G*”_ST_ and *D*_EST_ were considerably higher than *F*_ST_ (Tables E and F in S2 File), which is expected when within-population heterozygosity is high [61]. Overall, we detected significant differentiation between opposite roadsides and among same-roadside trap lines in all highways. The fact that there was a large overlap in the levels of genetic differentiation across comparisons supports the hypothesis that road surface avoidance is the main driver shaping the barrier effect for wood mice, and that significant genetic differentiation can develop within short periods after a highway has been built (Fig 1B*i*). Conversely, there was no evidence to support the hypothesis of genetic differentiation being mainly due to a traffic volume effect, as we did not find lower differentiation for AP51 (Fig 1A*i*) or a gradient of differentiation from AP51 to AP6 (Fig 1A*ii*).

**Fig 5:**
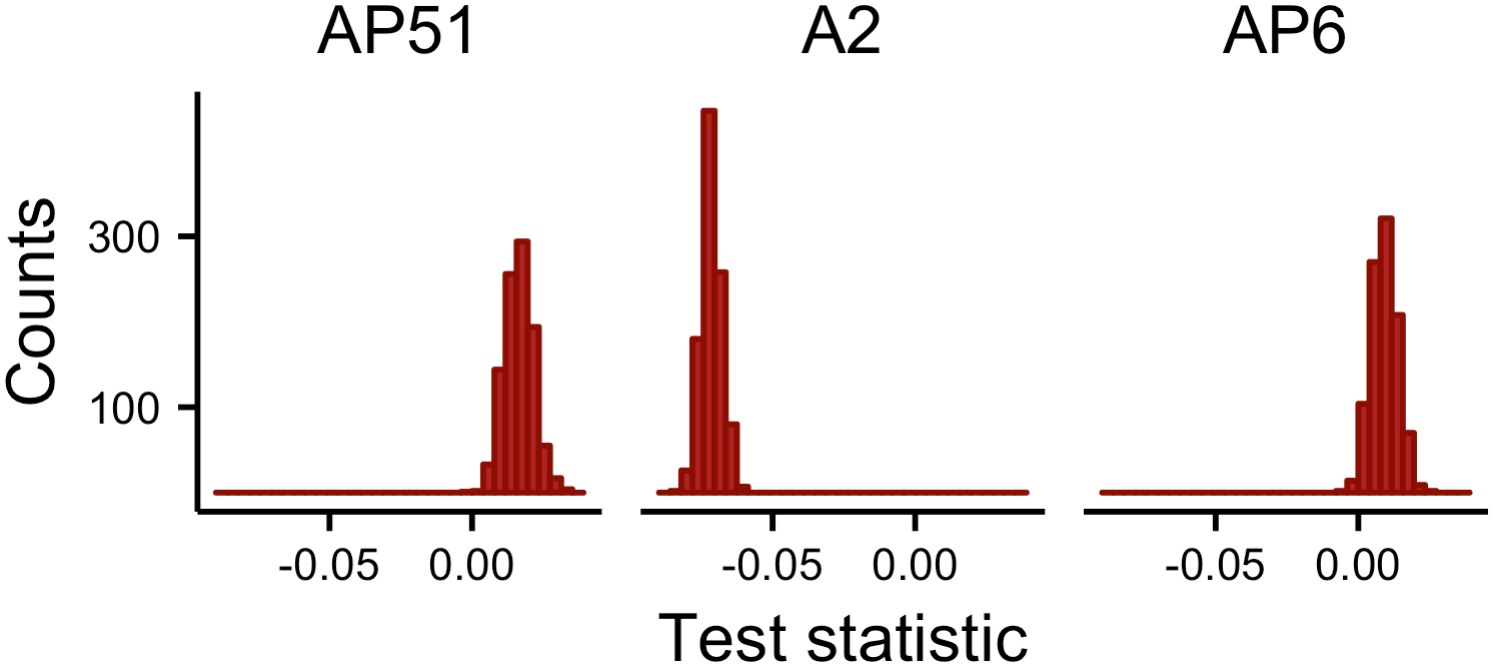
Distribution of *d*_GD_ based on *D*_c_ for each of the three highways. Positive values indicate higher average differentiation of samples between opposite roadsides relative to same-roadside samples. For AP6 and AP51, over 97% of the resamplings yielded test values greater than zero. For A2, all resamplings gave values less than zero.

The resampling-based test for differences in genetic distance *D*_c_ between opposite and same-roadside sampling lines provided evidence for higher average across-road differentiation for highways AP6 and AP51 (Fig 5). For highway A2, however, the genetic distance between same-roadside sampling lines was on average greater than between opposite sampling lines. Results using *F*_ST_ were similar (not shown).

### Estimating migration rates between opposite roadsides

The sensitivity analysis of the simulation model over the tested parameter ranges suggested that ‘Population size’ and ‘Same-roadside immigrants’ were the parameters that most significantly influenced *F*_ST_, *G”*_ST_, and *D*_EST_ (Fig A in S2 File). However, the parameter ‘Same-roadside immigrants’ only had a large effect under the scenario of complete isolation between roadsides, and in the case of *F*_ST_ also when the migration rate was 0.01. *G”*_ST_ and *D*_EST_, but not *F*_ST_, were also sensitive to the initial number of alleles, particularly when the across-road migration rate was ≤ 0.01. A higher sensitivity of *D*_EST_ to the initial level of genetic diversity as compared to *F*_ST_ has been observed previously [85].

**Fig 6:**
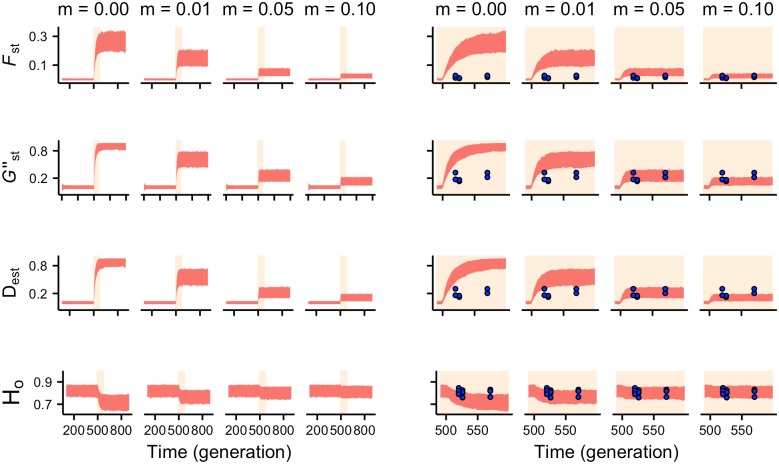
Values of *F*_ST_, *G”*_ST_, *D*_EST_ and *H*_o_ through time (generations) estimated from computer simulations for different migration rates after a ‘highway’ replaces at generation 500 a previous scenario of panmixia and assuming a multigeneration *Ne* of 54 for each roadside site. For each migration rate (m), the plots in the left panel show the evolution of the parameters through the simulation and the plots in the right panel show a detail of the simulations for the period in which the ‘highway’ has been introduced (apricot areas in the left plots). The 5–95 percentile envelopes (999 replicates for each migration rate value) are shown in light coral. In the right plots, the blue circles represent observed values, which are placed to the right of generation 500 according to the highway’s age and assuming a generation time of six months [88].

After ‘highway’ introduction, the increase in *F*_ST_, *G”*_ST_ and *D*_EST_ values was inversely proportional to the level of gene flow (Fig 6). The three estimators reached a new ‘equilibrium’ value relatively rapidly at between ten and 40 generations after ‘highway’ establishment. This result is in agreement with previous studies showing that at high mutation rates the time taken to reach 95% of the equilibrium value is similar for the three statistics [61,86], and that equilibrium can be reached faster when heterozygosity is high and *N*_e_ is not very large [85,87]. However, when the assumed migration rate was very low, *F*_ST_ took a longer time to reach ‘equilibrium’; this behavior of *F*_ST_ has been reported before [87]. Moreover, values of *G”*_ST_ and *D*_EST_ increased much faster than *F*_ST_, as in the simulations of [31] and [85], thereby facilitating earlier detection of the barrier. Observed values of *F*_ST_, *G”*_ST_ and *D*_EST_ were consistent with those estimated from simulations with a migration rate of 5–10% (Fig 6). In particular, the observed values of the latter two parameters closely matched the simulation results for a migration rate of 5%. As shown by the results of simulations assuming an *N*_e_ of 20 or 100 for each roadside site (Fig B in S2 File), so as to cover the range of the mean *N*_e_ estimates from LDNe for the 12 roadside sites (Table A in S2 File), the actual inferred migration rate depends on the assumed *N*_e_. The main point is that, for a plausible range of *N*_e_ values, the patterns of across-road genetic differentiation for the three highways are compatible with recurrent migration rates that are relatively low and, despite differences in age and traffic volume, similar among highways.

## Discussion

In this study we used different population genetic methods to investigate the presence and causes of the road barrier effect. We performed a comparative study of three highways of similar width but differing in age and traffic volumes, and used the wood mouse as a model species of forest-dwelling small mammals. The results of the different analyses provide consistent evidence for the presence of a highway barrier effect in the wood mouse apparently due to road surface avoidance. This conclusion is supported by the generally similar genetic patterns observed across the three studied highways. If road mortality or traffic avoidance were major components of the barrier effect, genetic differentiation between opposite roadsides should have been lowest for the highway with the lower traffic volume, but this was not evident in any of our analyses. Besides, if road age was a primary factor, we would expect genetic differentiation between opposite sides of the older highway to be significantly higher, but this was also not supported by our data (Figs 3 and 4). The hypothesis that best fits our results is that road surface avoidance is the primary cause of the barrier effect and that genetic responses to highways among wood mouse populations in our study area have short lag times (Fig 1B_*i*_). Other road genetic studies have demonstrated that genetic divergence can develop rather quickly when the barrier to gene flow is strong [9,21,68,89]. In each sampling site, the distance between trap lines was about 150 m and the maximum distance between traps was ca. 500 m. Since studies on wood mice have recorded dispersal movements of at least 300–500 m [90,91,92,93] and frequent excursions of 100–250 m beyond home range boundaries [90,94,95], the genetic differentiation observed between opposite trap lines is interpreted as due to the highway and not to isolation by distance [96]. In this context, we note a recent study reporting non-significant pairwise *F*_ST_ values of 0.008–0.014 between arable sample sites 1.5–6 Km apart in a farmland landscape intersected by roads and rivers [97]. Thus, even wood mice separated by much greater distances than those between opposite trap lines in our study are not necessarily significantly genetically differentiated. In addition, there is no evidence of previous barriers such as rivers or streams at the locations of the studied highway segments that could have caused the observed genetic differentiation. It is possible that the importance of traffic and road age is greater on older highways or those with higher traffic volumes than those studied here, and this hypothesis requires further investigation.

For AP6 and AP51, wood mice on opposite roadsides, despite being only about 150 m apart, were on average more genetically distinct than wood mice from sampling lines on the same side of the highway but more than 1 km apart and separated by viaducts (Figs 3 and 4). This result is revealing since one might have expected genetic differentiation to be greater among same-roadside samples given the distance between them and the results of previous studies of genetic structure in wood mice [83,84]. The lower across-road differentiation found in the A2 may be due to the higher number of culverts on the studied segment of this highway than on the segments of the other two highways (author’s observation). Wood mice are known to use highway crossing structures, including culverts [98], underpasses [99] and overpasses [100], and this may reduce the barrier effect. In agreement, on all three highways the genetic differentiation between opposite roadsides was lower at the sampling site with crossing structures nearby (Figs 3 and 4). Wood mice tend to avoid open terrain without ground cover that could offer protection from predators [24,101], and this has been invoked to explain fine-scale genetic structure [84] and inhibition of road crossings [6]. Our results indicate that highway passages may provide landscape connectivity for wood mice and reduce genetic differentiation between roadside populations.

The low *F*_ST_ values (0.008–0.028) could be interpreted as suggesting high migration rates between opposite sides of highways, but genetic differentiation measured by *F*_ST_ is constrained when within-population heterozygosity is high [102,103,104], which is the case for all our highway sites. [31] showed that the lag time for detection of a new barrier could be long using *F*_ST_, but much shorter using standardized *G*_ST_. Actually, the magnitude of standardized *G*_ST_ values we found between opposite roadsides is comparable to that obtained between populations 0.85–3 Km apart in a study of microgeographic structure in wood mice [84].

Similarly to our case, previous road genetic studies in small mammals have found non-significant or low *F*_ST_ values between highway sides: 0.018–0.048 [21]; 0.0018 [22]; and 0.005–0.036 but non-significant [23]. This may be due to the aforementioned deflation of *F*_ST_ with high subpopulation heterozygosity [21] or the lag time associated with *F*_ST_. This lag time should increase with larger *Ne*, and this reasoning has been used to explain the lack of genetic differentiation between common vole *Microtus arvalis* populations separated by a 25-year-old motorway [22]. In contrast, [21] asserted that the genetic differentiation they observed between bank vole *Myodes glareolus* populations separated by a highway of the same age could be expected because such time period corresponds to 25–50 generations of bank voles. In the common vole study, support for the inference of a very large *Ne* may be due to the assumption that the motorway led to two completely isolated populations, which might be an extreme scenario given the evidence of road crossing attempts by the species [105,106]. More generally, different results between studies on the effect of roads on genetic differentiation may be partly due to differences in study design and scale [9,68], with some studies sampling over relatively large areas of the landscape [22,107] and others sampling from both sides of individual roads [21,68].

Our LDNe estimates of *Ne* for the roadside sites ranged between 20 and 100 (Table A in S2 File); previous studies of genetic structure in wood mice also inferred small local *Ne* [84,97]. The linkage disequilibrium (LD) method implemented in LDNe can provide precise *Ne* estimates for relatively small populations (*Ne* < 200), is not likely to mistake a population with moderately small *Ne* for one with large *Ne* (and vice-versa), is robust to equilibrium immigration rates up to 0.1, and performs well in populations with skewed sex ratio or non-random variance in reproductive success [108,109,110]. However, the underlying model for the LD method assumes discrete generations; when samples are taken from age-structured species, the estimate from the LD method can be an estimate of the effective number of breeders (*Nb*) that produced the cohort(s) from which the sample was taken or of a quantity intermediate between *Nb* and *Ne* [108,111]. Recent work suggests that estimates from the LD method based on random samples of adults may be on average 20–30% lower than the true *Ne* per generation, but the downward bias tends to be less in species in which the reproductive lifespan approaches the generation length [112] (see below). In our simulation, we assumed a constant (moderately small) *Ne* within the range of the LDNe estimates across sampling lines. The rationale for this choice was that, given our estimates of contemporary *Ne* and the large annual population fluctuations of wood mice [33,113], the multigeneration *Ne* may be expected to be moderate [114,115]. If this *Ne* is larger than we assumed, as possibly indicated by the values of *F*_ST_ (<0.05) and *D*_EST_ (>0.1) between opposite sides of the highways (Table E in S2 File) [85], then the migration rate may be lower than that inferred from the simulation [e.g. 89]. A strong road barrier effect combined with a moderate multigeneration *Ne* may explain why we were able to detect significant across-road genetic differentiation after 20 generations of wood mice. It could be argued that the fact that we detected differentiation even for the newest highways (ca. 10-years-old) could be due to the relatively small roadside samples used in this study [116,117], but the inference of a road barrier effect is supported by the greater average differentiation observed between individuals from opposite roadsides than between those more than 1 Km apart on the same roadside (Fig 5). Moreover, another study on wood mice, with sample sizes per site and number of microsatellite markers used similar to those in ours, found greater average differentiation between urban than arable sites, with the latter for the most part not significantly differentiated from each other [97]. However, future research, sampling more individuals along more extensive stretches of the highway verges, is needed to confirm our results.

In agreement with previous genetic studies pertaining to roads, which generally found reduced but ongoing gene flow between opposite roadside populations (reviewed in [9], our simulations indicated limited connectivity across the highways. Combining empirical and simulation analyses can be a powerful approach in landscape genetic studies [118,119]. Here, we used a simple simulation model that yielded *F*_ST_, *G”*_ST_ and *D*_EST_ values compatible with observed ones when the assumed across-road migration rate in the simulations was about 0.05. These results further support the conclusion that gene flow is restricted between roadsides and of the same level across the studied highways, despite differences in age and traffic volume, so that they exhibit similar genetic patterns. The inferred gene flow may be the result of the numerous regularly spaced culverts in the studied highways, but given the relatively low migration rate estimated, which could be even lower if the local *Ne* of the roadside populations are larger than we assumed (Fig B in S2 File), such passages are perhaps not effectively counteracting the road barrier effect. Nevertheless, we highlight that the surveyed sites without passages had higher, albeit not significantly different, genetic differentiation. This suggests that crossing structures may indeed improve the permeability of highways to wood mice movement. However, a significant interchange of individuals per generation may be needed to prevent substantial population differentiation [120,121], for example when the ratio of effective population size to census population size (*Ne*/*N*) is small [122, 123] or when migrants are likely to be at disadvantage in terms of survival and breeding success, as in the case of territorial species [118].

The most subjective parameter in the simulations was related to the number of immigrants received in each generation from neighboring populations on the same side of the highway. This parameter was included in the model because it is unlikely that roadside populations are isolated from other populations on the same side of the highway and, since observed individuals were highly polymorphic, to prevent fixation of alleles during simulations. We assumed that, for each roadside and in each generation, a single immigrant replaced a resident. The sensitivity analysis showed that variation in this parameter would significantly affect model predictions for *H*_o_. However, increasing the number of immigrants raised the values of *H*_o_ (Fig A in S2 File) so that their averages were more dissimilar from those of the observed data (Table D in S2 File). This further suggests that the model assuming one same-roadside immigrant into each roadside population could accurately infer the most probable maximum migration rate between opposite roadsides. Our simulations contained a number of simplifying assumptions regarding life history and demographic traits, including sex ratio, mating process, breeding frequency, and population size. We are not aware of specific estimates of generation time for wood mice. The maximum longevity in the wild is 15–20 months [124,125], and some studies found few individuals living for more than 12 months [126,127,128]. Generation time is often approximated by considering maximum longevity in the wild and age at first breeding, since the latter sets the generation time [129]. The age at first birth in the wood mouse is three months [130] and generation times of similar length have been estimated for other small rodents [131]. However, there is individual, seasonal, habitat-related and geographical variation in the age of sexual maturity [125,132,133,134], and the onset of sexual activity in females as a function of age can be delayed and slow [24,135]. Taking all this information together, we assumed a generation time of six months [88]. We also assumed an equal sex ratio. A slightly male-biased sex ratio (≈ 55:45, [125]) has been reported by several authors [133,136,137,138], while others have found a surplus of females among reproductive individuals [135,139]. In general, it is known that the sex ratio fluctuates yearly and seasonally, but it tends to become balanced before the start of the breeding season [125,136,137]. We assumed one reproductive event per individual because, although females can produce up to four litters annually [130], most (≈ 90%) breed only once or twice in their lifetime [24]. Wood mouse population dynamics is also complex with, for instance, seasonal and annual population fluctuations due to density-dependent factors and food availability [113,137,138,140]. Although we believe that our simulation could capture the main population dynamics and replicate the genetic patterns among wood mouse populations separated by barriers of varying permeability, future work should aim to develop more complex models that more realistically incorporate the biology and ecology of the species, including the occurrence of road mortality [99,141,142].

The importance of comparing genetic and ecological data in understanding population structure and connectivity has been repeatedly highlighted [21,118,143]. Our results, concerning both the inferred reduced gene flow and the lack of observed road crossings in the recapture data, concur with the findings of road ecology studies on wood mice [39]. For example, [40] recorded only four road crossings in a total of 994 recaptures (576 individuals) and [6] found that only six among 98 marked individuals (559 recaptures) crossed minor rural roads. Unpublished capture-mark-recapture data gathered by one of us (FA; sampling design described in [32]) also indicate that wood mice rarely crossed highway A2, with only five crossings between verges and the median strip in 251 recaptures of 124 individuals. However, direct measures can overestimate gene flow since dispersing individuals may not breed [118], or underestimate migration as they only record the sampling period and may miss seasonal or other movements [144]. Thus, comprehensive genetic analyses are invaluable in providing reliable estimates of gene flow and population differentiation (e.g. [68,89,107]).

This study provides evidence for the presence of a highway barrier effect in the wood mouse apparently due to road avoidance. This is likely an expression of behavioral avoidance of open areas [5,6,16,101] and not as a result of an inhibition to cross paved roads, as both attempted and successful paved road crossings have been recorded [39,40,99,141]. A road barrier effect due to road clearance avoidance is likely to occur in other small forest mammals [6,16,17,145]. The wood mouse is abundant, widely distributed, and not considered of conservation concern, but it is still important to maintain genetic cohesion among populations, particularly given the high density of the road network across its range. Thus, management measures that have been proposed to increase across-highway connectivity for small mammals, and also for other wildlife [146,147], would be worth considering. Although the effectiveness of crossing structures for maintaining genetic connectivity needs to be evaluated and monitored in the long term, they remain one of the few viable options for mitigating the effects of highways on small to medium-sized species [5,148,149]. One form of mitigation could be retrofitting of culverts and underpasses to increase their attractiveness and facilitate their use as highway crossings for wood mice and many other species. The use of culverts by wood mice and other small mammals has been reported in previous research [98,146,147], and our work also suggests a positive effect of these structures. Also, roadkill studies have found lower mortality of wood mice in road sections with culverts and underpasses [99,150]. Small mammals may prefer small culverts than underpasses with larger diameters and may preferentially use passages with abundant vegetation cover at entrances [17, 148,151]. Moreover, the retrofitting of road passages should ideally be undertaken in areas where verges have a mature vegetation structure, as they are known to provide refuge habitats and corridors for small mammals [32,33,152]. Accordingly, we suggest that mowing of verges, which is used to prevent vegetation from encroaching on the roadway and for traffic safety reasons, could be limited to a certain distance (ca. 10–12 m) from the pavement edge, with the rest of the verge managed to promote growth of tall and dense vegetation [16,17,32,33]. These roadside vegetation strips would facilitate establishment and dispersal of small mammals.

In conclusion, here we used the wood mouse as a model in a study designed to disentangle the causes of the road barrier effect and investigate the role of road avoidance through the analysis of genetic patterns, and the results contribute to our understanding of how small mammal populations may be affected by highways [153]. Moreover, it adds to the growing literature demonstrating that roads can have a rapid effect on the genetic connectivity and substructuring of wildlife populations.

## Supporting Information

S1 File Microsatellite genotypes. Microsatellite genotypes of the 302 wood mice samples arranged by trap line in GENALEX format.
(XLSX)

S2 File Tables A-F, Figs A-B. Estimates of effective population size using LDNe and ONeSAMP (**Table A**). Original and adjusted p-values for the HWE test (**Table B**). Loci with possible null alleles identified by MICRO-CHECKER (**Table C**). Estimates of genetic diversity for each trap line (**Table D**). Estimates of genetic differentiation between opposite highway sides for each of the three studied highways (**Table E**). Estimates of genetic differentiation between same-roadside sampling lines for the three studied highways (**Table F**). Sensitivity analyses of *F*_ST_, *G”*_ST_, *D*_EST_ and *H*_o_ to variation in the model parameters: ‘Population size’, ‘Initial alleles’, ‘Mutation model’, ‘Mutation rate’ and ‘Same-roadside immigrants’ (**Fig A**). Values of *F*_ST_, *G”*_ST_, *D*_EST_ and *H*_o_ through time (generations) estimated from computer simulations for different migration rates after a ‘highway’ replaces at generation 500 a previous scenario of panmixia and assuming a multigeneration *Ne* of 20 or 100 for each roadside site (**Fig B**).
(PDF)

S3 File NetLogo model. Code for the simulation model.
(NLOGO)
