# Supplementary material for: Disentangle the Causes of the Road Barrier Effect in Small Mammals through Genetic Patterns
Source: PLoS One. 2016 Mar 15;11(3):e0151500. doi: 10.1371/journal.pone.0151500 (PMC4792435; doi:10.1371/journal.pone.0151500)
Supplement: S2 File — Estimates of effective population size using LDNe and ONeSAMP (Table A). Original and adjusted p-values for the HWE test (Table B). Loci with possible null alleles identified by MICRO-CHECKER (Table C). Estimates of genetic diversity for each trap line (Table D). Estimates of genetic differentiation between opposite highway sides for each of the three studied highways (Table E). Estimates of genetic differentiation between same-roadside sampling lines for the three studied highways (Table F). Sensitivity analyses of FST, G”ST, DEST and Ho to variation in the model parameters: ‘Population size’, ‘Initial alleles’, ‘Mutation model’, ‘Mutation rate’ and ‘Same-roadside immigrants’ (Fig A). Values of FST, G”ST, DEST and Ho through time (generations) estimated from computer simulations for different migration rates after a ‘highway’ replaces at generation 500 a previous scenario of panmixia and assuming a multigeneration Ne of 20 or 100 for each roadside site (Fig B). (PDF) [file pone.0151500.s002.pdf]

Supporting information S2

Prior to the simulations, we estimated effective population size (N_e) using the program LDNe 1.31 [1], which corrects the bias in the linkage disequilibrium method when sample size is less than the true effective size [2,3]. This method can provide precise N_e estimates for relatively small populations [4] and is robust to equilibrium immigration rates up to 0.1 [5] and to sample size [6]. We used a model with random mating and, following the recommendations of [4], excluded alleles with frequencies less than 0.03. Parametric 95% confidence intervals (CIs) were computed using equation 12 in [3]. We also estimated N_e with ONeSAMP 1.2 [7]; a program that uses approximate Bayesian computation to compare summary statistics between a large number of simulated populations and the study population of interest. For all sampling lines, the lower and upper bounds of the prior on N_e were two and 100, respectively. Upper bounds of 200 and 500 were also tested to assess the sensitivity of the results to the choice of prior. Three replicate runs were carried out for each trap line.

The LDNe estimates of N_e for each sampling line were broadly similar across highways and sites (mean 54±22 individuals), with largely overlapping CI (Table A). Results from ONeSAMP for all highway verges centered around 20 (Table A), but they were sensitive to the upper bound of the prior on N_e, rapidly decreasing as the upper bound increased. The similarity of the ONeSAMP estimates across roadsides and their very narrow CI given the sample sizes suggests that these results should be considered with caution. The ONeSAMP method has been reported to be correlated with and sensitive to sample size in certain datasets [6,8,9]. However, the results from ONeSAMP were within the range of N_e values tested in the sensitivity analysis of computer simulations (see Table 1 in main text).

Table A. Estimates of effective population size using LDNe and ONeSAMP. Intervals within square brackets are 95% confidence intervals.

Site	Trap Line	LDNe		ONeSAMP	
A2_1	E	64	[45-106]	23	[21-24]
	W	72	[47-142]	20	[19-22]
A2_2	E	55	[39-87]	22	[20-23]
	W	39	[29-54]	22	[21-24]
AP51_1	N	44	[32-67]	19	[18-20]
	S	103	[61-275]	23	[22-25]
AP51_2	N	43	[30-69]	14	[13-15]
	S	70	[47-128]	23	[21-25]
AP6_1	E	39	[29-56]	15	[14-16]
	W	69	[47-121]	23	[22-25]
AP6_2	E	21	[17-27]	18	[17-19]
	W	30	[24-40]	17	[16-18]

Table B. Original and adjusted p-values for the HWE test. Loci that remained significant after sequential Bonferroni correction are indicated in pink. Results are for trap line on each roadside (East or West, North or South) of the highway sampling sites.

**A2**	**Site 1**		**Site 2**	
**Loci**	E	W	E	W
AS7	0.561/1.000	0.422/1.000	0.089/0.356	0.873/0.873
AS11	0.000/0.000	0.000/0.000	0.029/0.174	0.006/0.054
AS20	0.953/1.000	0.980/1.000	0.155/0.465	0.041/0.246
AS34	0.952/1.000	0.983/1.000	0.000/0.000	0.022/0.154
SCFM2	0.020/0.160	0.081/0.486	0.290/0.580	0.010/0.080
SCFM6	0.045/0.270	0.001/0.008	0.003/0.021	0.080/0.400
SCFM9	0.027/0.189	0.001/0.008	0.002/0.016	0.083/0.400
SFM2	0.719/1.000	0.800/1.000	0.043/0.215	0.274/0.548
TNF	0.087/0.435	0.418/1.000	0.431/0.580	0.149/0.447
**AP51**	**Site 1**		**Site 2**	
**Loci**	N	S	N	S
AS7	0.020/0.100	0.000/0.000	0.242/1.000	0.363/0.801
AS11	0.000/0.000	0.005/0.035	0.137/1.000	0.001/0.008
AS20	0.044/0.176	0.211/0.844	0.959/1.000	0.267/0.801
AS34	0.000/0.000	0.037/0.185	0.000/0.000	0.038/0.228
SCFM2	0.177/0.531	0.000/0.000	0.176/1.000	0.000/0.000
SCFM6	0.569/0.634	0.359/0.844	0.265/1.000	0.155/0.620
SCFM9	0.013/0.078	0.224/0.844	0.244/1.000	0.021/0.147
SFM2	0.001/0.007	0.030/0.180	0.855/1.000	0.364/0.801
TNF	0.317/0.634	0.747/0.844	0.190/1.000	0.072/0.360
**AP6**	**Site 1**		**Site 2**	
**Loci**	E	W	E	W
AS7	0.409/1.000	0.047/0.329	0.010/0.070	0.001/0.008
AS11	0.000/0.000	0.002/0.018	0.026/0.130	0.058/0.290
AS20	0.003/0.021	0.076/0.380	0.303/0.606	0.015/0.090
AS34	0.198/0.990	0.105/0.380	0.000/0.000	0.000/0.000
SCFM2	0.964/1.000	0.104/0.380	0.012/0.072	0.005/0.035
SCFM6	0.427/1.000	0.027/0.216	0.001/0.008	0.076/0.304
SCFM9	0.472/1.000	0.079/0.380	0.830/0.830	0.615/0.615
SFM2	0.000/0.000	0.886/0.886	0.181/0.604	0.195/0.585
TNF	0.142/0.852	0.047/0.329	0.151/0.604	0.273/0.585

Table C. **Loci with possible null alleles identified by MICRO-CHECKER.** Within parentheses are the null allele frequencies estimated by ML-NullFreq. Results are for trap line on each roadside (East or West, North or South) of the highway sampling sites.

**A2**			
**Site 1**		**Site 2**	
**E**	**W**	**E**	**W**
AS11 (0.17)<br>SCFM9 (0.08)	AS11 (0.20)<br>SCFM6 (0.13)<br>SCFM9 (0.11)	AS34 (0.15)<br>SCFM6 (0.10)<br>SCFM9 (0.11)	AS11 (0.10)<br>SCFM2 (0.09)

**AP51**			
**Site 1**		**Site 2**	
**N**	**S**	**N**	**S**
AS11 (0.17)<br>AS34 (0.17)	AS7 (0.14)<br>AS11 (0.07)<br>SCFM2 (0.15)	AS34 (0.19)	AS11 (0.12)<br>SCFM2 (0.15)

**AP6**			
**Site 1**		**Site 2**	
**E**	**W**	**E**	**W**
AS11 (0.09)<br>SFM2 (0.22)	AS11 (0.08)	AS7 (0.10)<br>AS34 (0.20)<br>SCFM2 (0.11)<br>SCFM6 (0.14)	AS7 (0.09)<br>AS34 (0.14)<br>SCFM2 (0.09)

Table D. Estimates of genetic diversity for each trap line. n, number of individuals; A, average number of alleles across loci; H_o, average observed heterozygosity; H_e, average expected heterozygosity; F_{IS}, inbreeding coefficient; R_{QG}, mean pairwise relatedness; P_A, number of private alleles (relative to the sampling line in the opposite roadside).

	Site 1		Site 2	
**A2**	**E**	**W**	**E**	**W**
n	26	25	25	25
A	16.8	16.0	15.6	14.1
H_o	0.85±0.13	0.81±0.16	0.76±0.08	0.82±0.08
H_e	0.93±0.02	0.92±0.02	0.92±0.03	0.92±0.02
F_{IS}	0.09 [0.01-0.12]	0.12 [0.05-0.16]	0.17 [0.07-0.23]	0.11 [0.04-0.14]
R_{QG}	0.02 [0.01-0.03]	0.03 [0.01-0.04]	0.02 [0.01-0.04]	0.03 [0.01-0.04]
P_A	10	6	16	12
**AP51**	**N**	**S**	**N**	**S**
n	25	25	23	27
A	16.4	16.2	14.7	16.0
H_o	0.80±0.15	0.81±0.12	0.82±0.12	0.79±0.11
H_e	0.92±0.02	0.92±0.03	0.90±0.03	0.91±0.02
F_{IS}	0.13 [0.05-0.17]	0.12 [0.05-0.15]	0.09 [0.02-0.13]	0.14 [0.07-0.17]
R_{QG}	0.02 [0.00-0.03]	0.02 [0.01-0.03]	0.05 [0.03-0.06]	0.04 [0.03-0.05]
P_A	5	10	12	8
**AP6**	**E**	**W**	**E**	**W**
n	24	27	25	25
A	14.6	17.0	13.3	15.2
H_o	0.82±0.14	0.83±0.05	0.76±0.14	0.83±0.13
H_e	0.92±0.02	0.92±0.03	0.90±0.03	0.91±0.02
F_{IS}	0.11 [0.04-0.13]	0.10 [0.02-0.14]	0.16 [0.08-0.20]	0.10 [0.02-0.13]
R_{QG}	0.03 [0.01-0.04]	0.02 [0.01-0.03]	0.06 [0.04-0.07]	0.03 [0.01-0.05]
P_A	7	9	9	15

Table E. Estimates of genetic differentiation between opposite highway sides for each of the three studied highways. F_{ST}, F_{ST} (ENA), G''_{ST}, and D_{EST} are, respectively, the genetic differentiation estimators of [10], using the ENA (excluding null alleles) correction of [11], of [12], and of [13]. D_c is the chord genetic distance [14]. Intervals within square brackets are 95% confidence intervals. For the G-test [15], numbers are *p-values* obtained using, respectively, the Fisher's method and a generalized binomial test to combine results over loci.

**Parameter**	**A2_1**	**A2_2**	**AP51_1**	**AP51_2**	**AP6_1**	**AP6_2**
F_{ST}	0.008 [0.000-0.016]	0.011 [0.004-0.017]	0.012 [0.004-0.021]	0.027 [0.017-0.038]	0.016 [0.008-0.026]	0.028 [0.017-0.042]
F_{ST} (ENA)	0.007 [0.000-0.015]	0.01 [0.004-0.017]	0.009 [0.003-0.018]	0.027 [0.018-0.037]	0.016 [0.008-0.026]	0.025 [0.016-0.036]
G''_{ST}	0.107 [0.001-0.211]	0.133 [0.046-0.244]	0.146 [0.055-0.259]	0.304 [0.199-0.402]	0.2 [0.103-0.304]	0.302 [0.192-0.444]
D_{EST}	0.1 [0.001-0.198]	0.124 [0.042-0.231]	0.136 [0.051-0.244]	0.284 [0.186-0.380]	0.187 [0.096-0.287]	0.282 [0.178-0.420]
D_c	0.402 [0.350-0.463]	0.429 [0.366-0.493]	0.415 [0.362-0.470]	0.471 [0.437-0.509]	0.429 [0.382-0.479]	0.466 [0.414-0.529]
G-test	0.006/0.026	0.000/0.000	0.002/0.047	0.000/0.000	0.000/0.000	0.000/0.000

Table F. Estimates of genetic differentiation between same-roadside sampling lines for the three studied highways. F_{ST}, F_{ST} (ENA), G''_{ST}, and D_{EST} are, respectively, the genetic differentiation estimators of [10], using the ENA (excluding null alleles) correction of [11], of [12], and of [13]. D_c is the chord genetic distance [14]. Intervals within square brackets are 95% confidence intervals. For the G-test [15], numbers are *p-values* obtained using, respectively, the Fisher's method and a generalized binomial test to combine results over loci.

**Parameter**	**A2_E**	**A2_W**	**AP51_N**	**AP51_S**	**AP6_E**	**AP6_W**
F_{ST}	0.015 [0.003-0.029]	0.015 [0.001-0.031]	0.019 [0.010-0.028]	0.015 [0.008-0.023]	0.023 [0.017-0.029]	0.011 [0.005-0.017]
F_{ST} (ENA)	0.015 [0.003-0.028]	0.014 [0.001-0.029]	0.019 [0.013-0.026]	0.014 [0.006-0.021]	0.024 [0.018-0.031]	0.01 [0.004-0.016]
G''_{ST}	0.205 [0.034-0.389]	0.187 [0.014-0.414]	0.227 [0.131-0.308]	0.18 [0.096-0.260]	0.251 [0.182-0.332]	0.14 [0.063-0.217]
D_{EST}	0.192 [0.031-0.372]	0.174 [0.013-0.395]	0.212 [0.122-0.290]	0.167 [0.088-0.243]	0.233 [0.167-0.314]	0.13 [0.059-0.204]
D_c	0.456 [0.360-0.563]	0.473 [0.384-0.585]	0.429 [0.375-0.488]	0.423 [0.380-0.465]	0.477 [0.420-0.537]	0.391 [0.319-0.467]
G-test	0.000/0.000	0.000/0.000	0.000/0.000	0.000/0.002	0.000/0.000	0.000/0.001

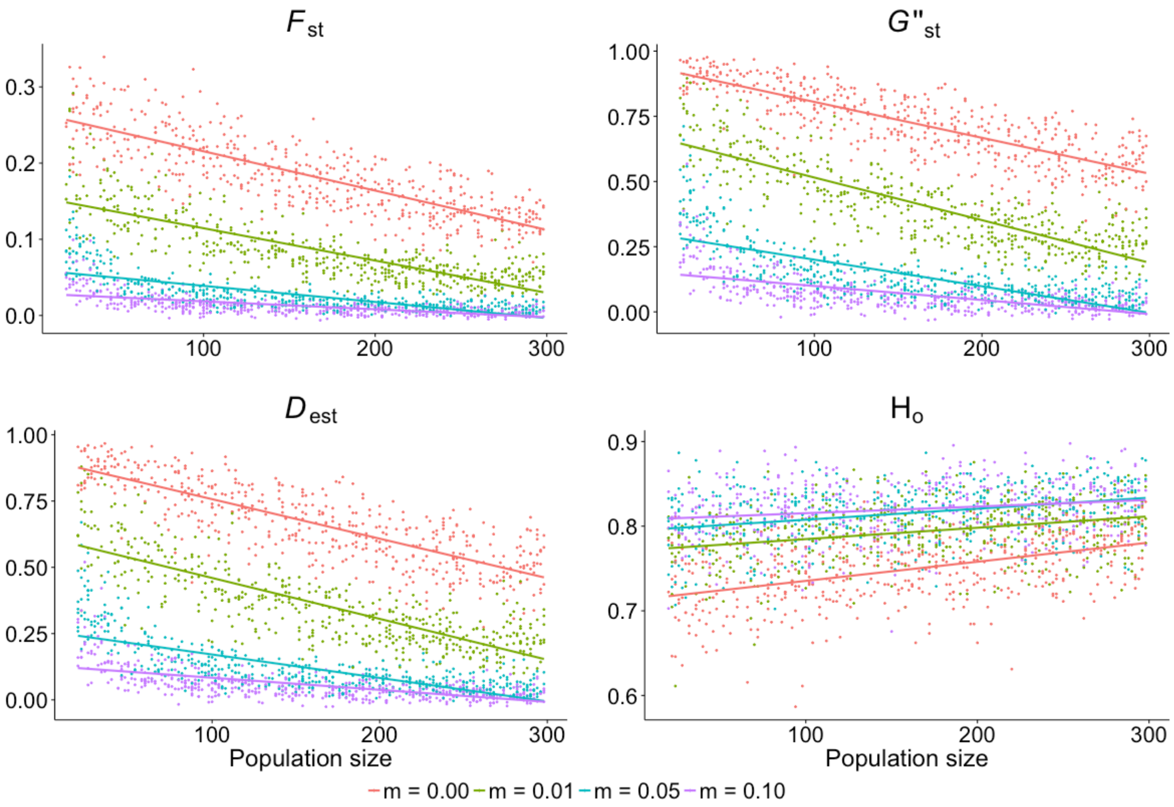
F_{st}
G''_{st}
D_{est}
H_o
Population size
Population size
m = 0.00
m = 0.01
m = 0.05
m = 0.10

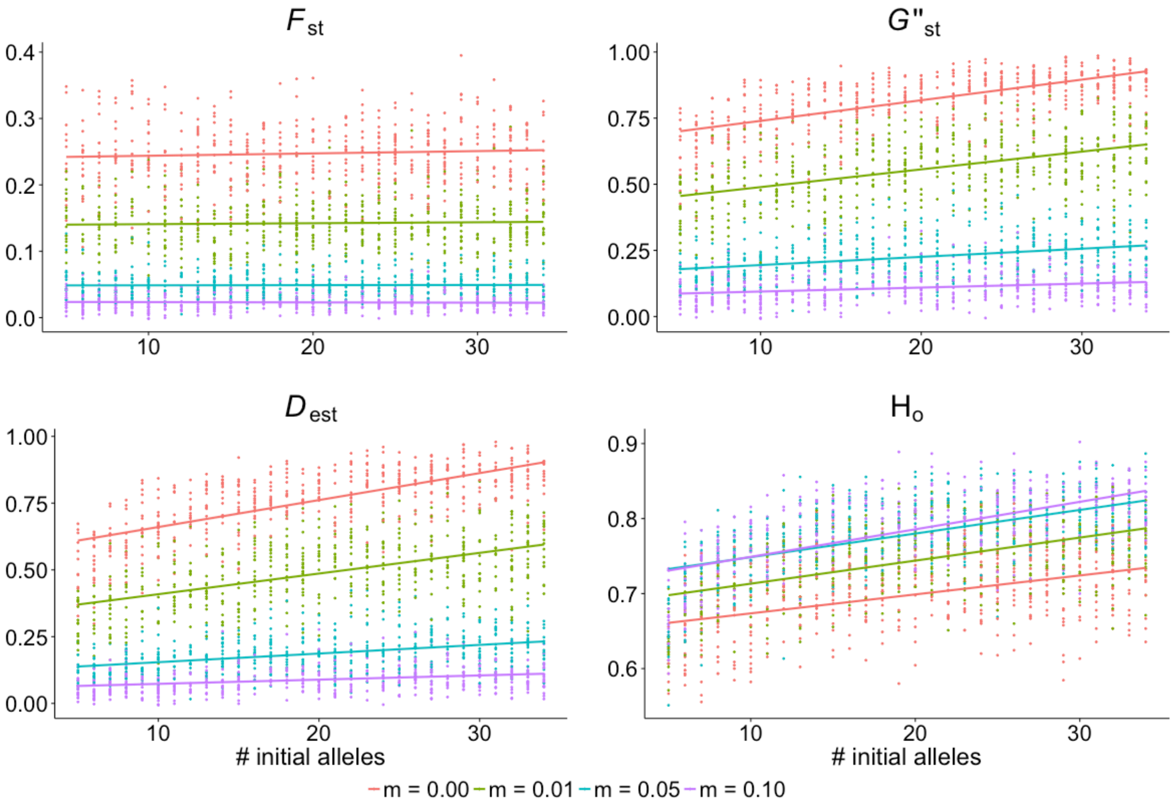
F_{st}
G''_{st}
D_{est}
H_o
# initial alleles
# initial alleles
m = 0.00
m = 0.01
m = 0.05
m = 0.10

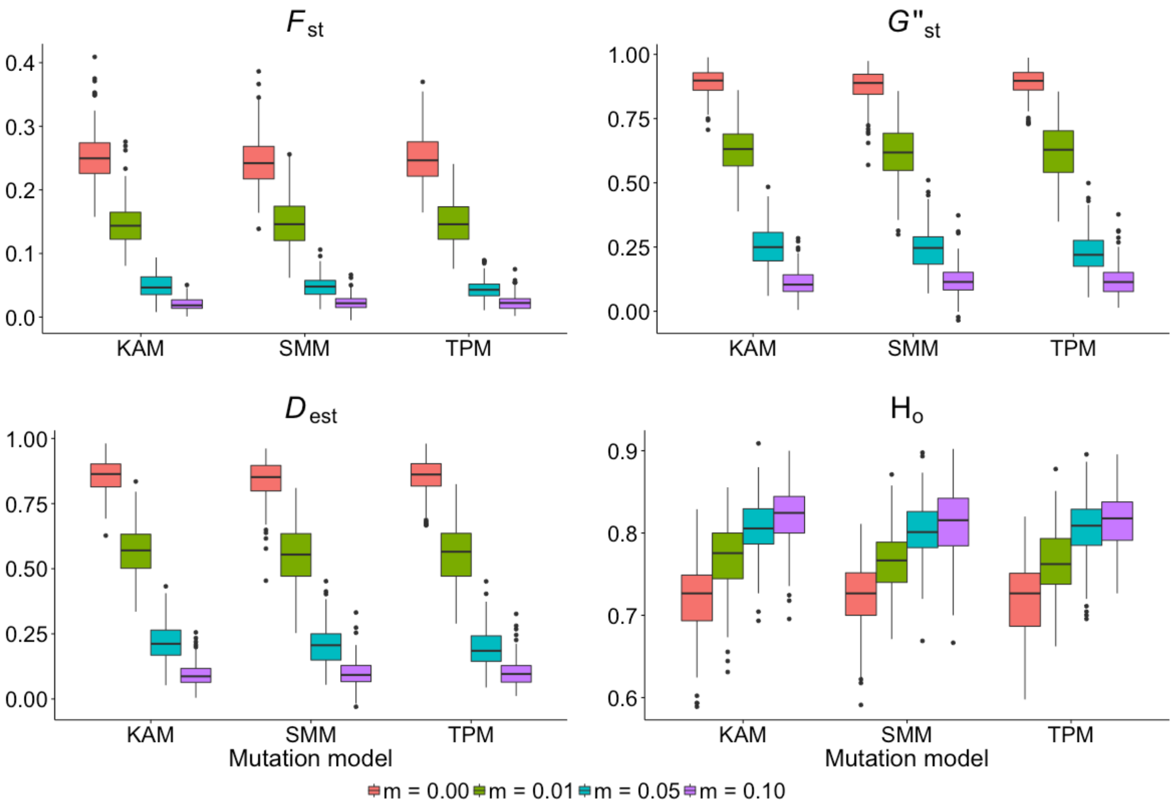

Fst
G"st
Dest
Ho
KAM
SMM
TPM
Mutation model
m = 0.00
m = 0.01
m = 0.05
m = 0.10

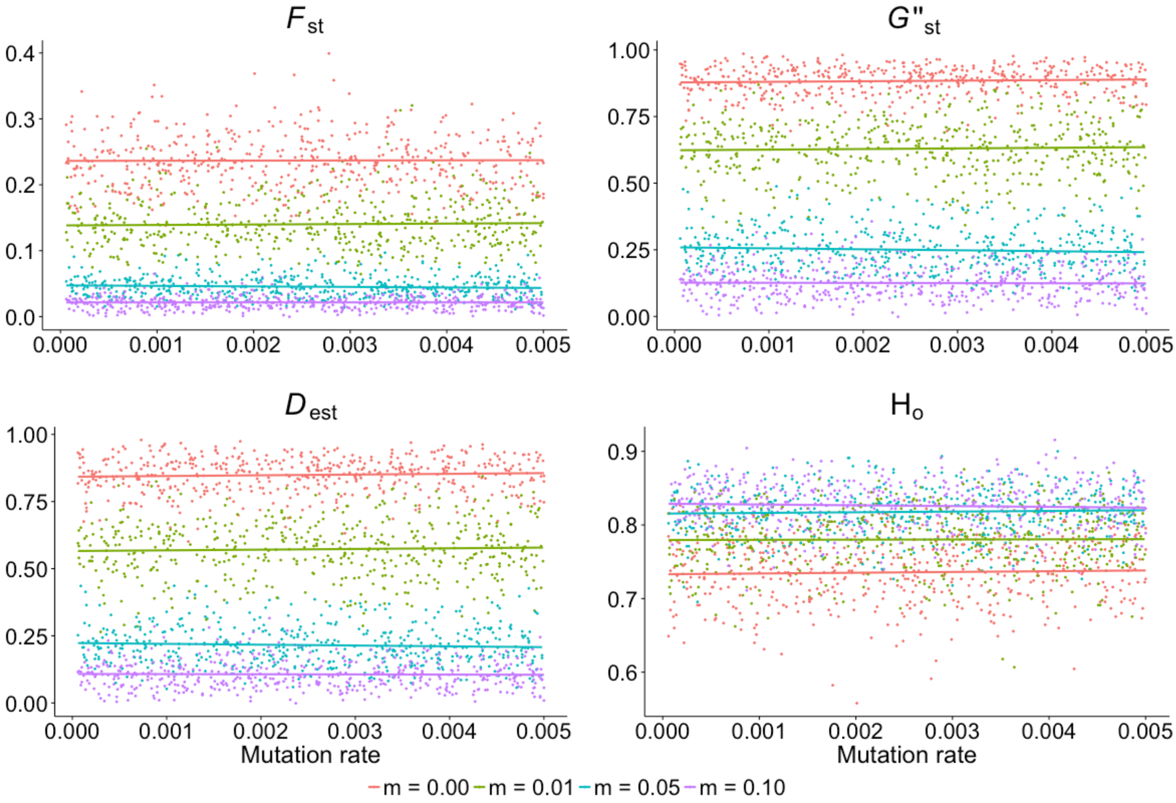

Fst
G"st
Dest
Ho
Mutation rate
m = 0.00
m = 0.01
m = 0.05
m = 0.10

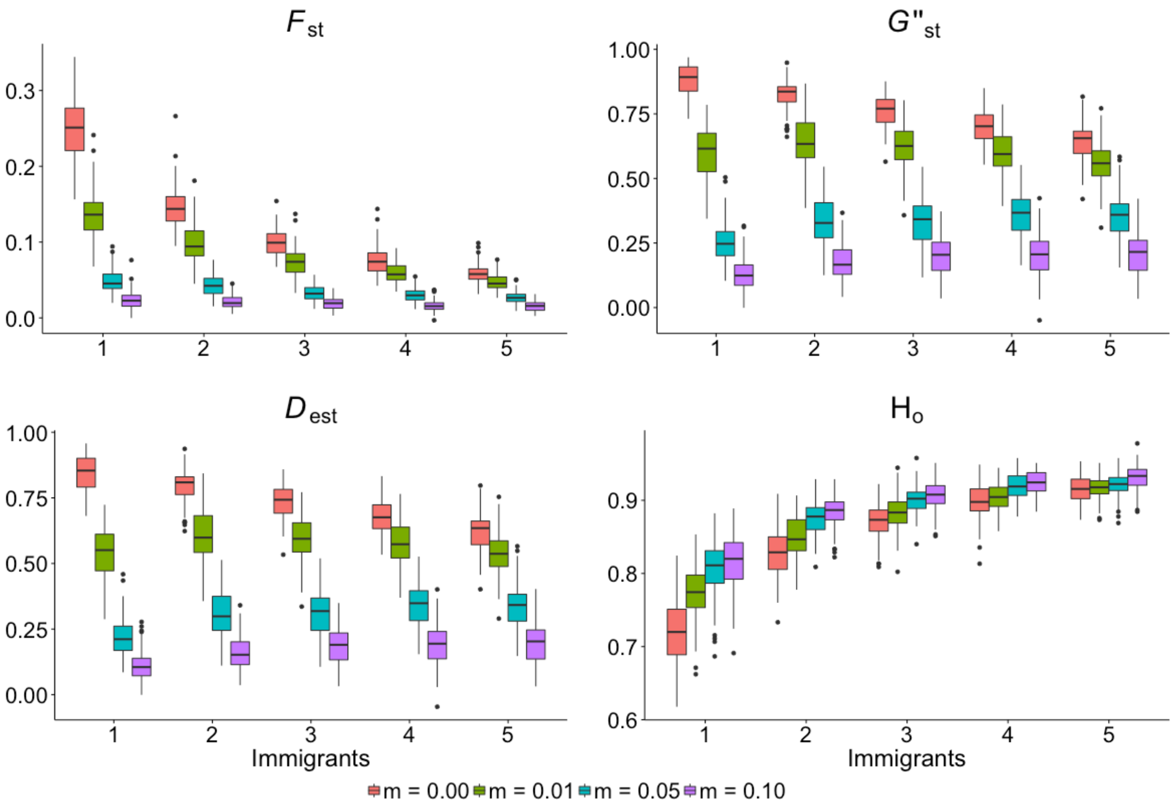

Figure A. Sensitivity analyses of F_{ST}, G''_{ST}, D_{EST} and H_o to variation in the model parameters: 'Population size', 'Initial alleles', 'Mutation model', 'Mutation rate' and 'Same-roadside immigrants'. The four colors represent four different across-road migration rate (m) scenarios. In the plots concerning 'Population size', 'Initial alleles' and 'Mutation rate', the dots represent estimates from 500 simulations for each migration rate value and the lines are regression lines. The graphs for the parameters 'Mutation model' and 'Same-roadside immigrants' are Tukey box plots.

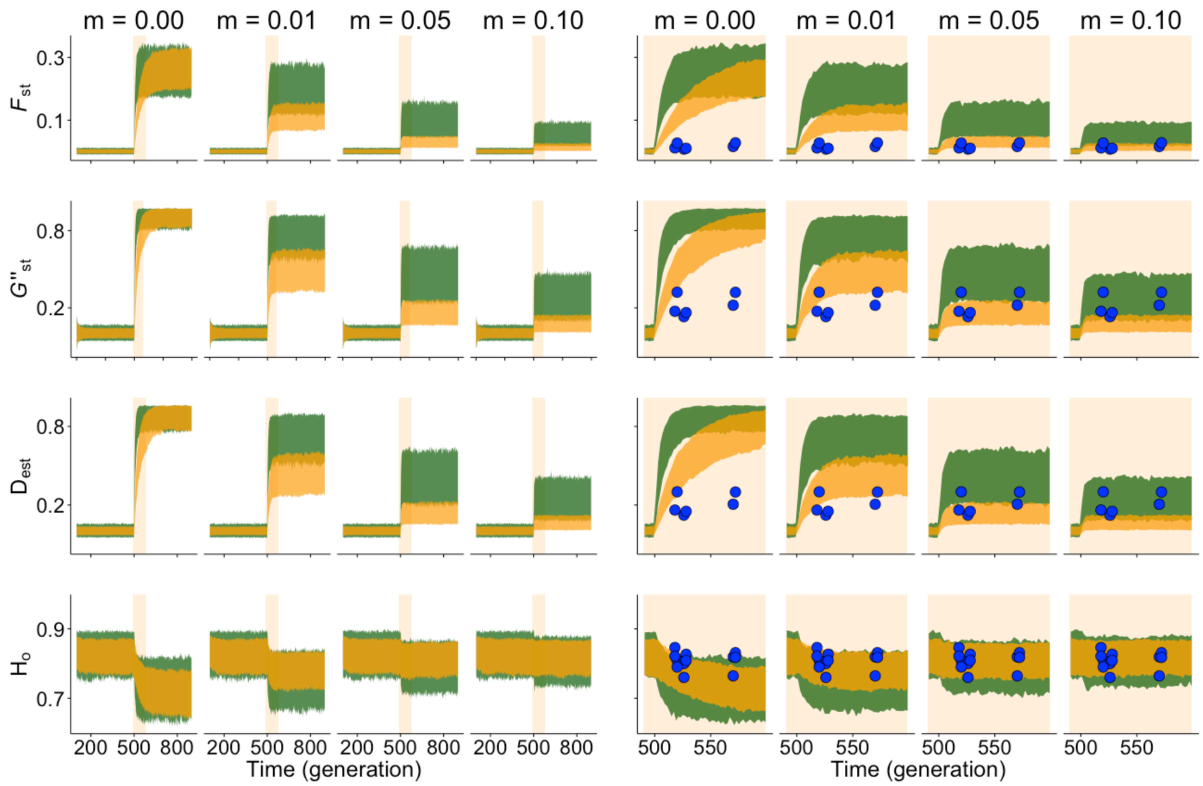

Figure B. Values of F_{ST}, G''_{ST}, D_{EST} and H_o through time (generations) estimated from computer simulations for different migration rates after a 'highway' replaces at generation 500 a previous scenario of panmixia and assuming a multigeneration *Ne* of 20 or 100 for each roadside site. For each migration rate (m), the plots in the left panel show the evolution of the parameters through the simulation and the plots in the right panel show a detail of the simulations for the period in which the 'highway' has been introduced (apricot areas in the left plots). The 5-95 percentile envelopes (999 replicates for each migration rate value) are shown in green (*Ne* = 20) and in orange (*Ne* = 100). In the right plots, the blue circles represent observed values, which are placed to the right of generation 500 according to the highway's age and assuming a generation time of six months [16].

.

References

1. Waples RS, Do C (2008) LDNe: a program for estimating effective population size from data on linkage disequilibrium. Mol Ecol Resour 8: 753–756.

2. England PR, Cornuet JM, Berthier P, Tallmon DA, Luikart G (2006) Estimating effective population size from linkage disequilibrium: severe bias in small samples. Conserv Genet 7: 303–308.

3. Waples RS (2006) A bias correction for estimates of effective population size based on linkage disequilibrium at unlinked gene loci. Conserv Genet 7: 167–184.

4. Waples RS, Do C (2010) Linkage disequilibrium estimates of contemporary Ne using highly variable genetic markers: a largely untapped resource for applied conservation and evolution. Evol Appl 3: 244–262.

5. Waples RS, England PR (2011) Estimating contemporary effective population size on the basis of linkage disequilibrium in the face of migration. Genetics 189: 633–644.

6. Haag T, Santos AS, Sana DA, Morato RG, Cullen Jr L, et al. (2010) The effect of habitat fragmentation on the genetic structure of a top predator: loss of diversity and high differentiation among remnant populations of Atlantic Forest jaguars (Panthera onca). Mol Ecol 19: 4906–4921.

7. Tallmon DA, Koyuk A, Luikart G, Beaumont MA (2008) ONeSAMP: a program to estimate effective population size using approximate Bayesian computation. Mol Ecol Resour 8: 299–301.

8. Sotelo G, Morán P, Fernández L, Posada D (2008) Genetic variation of the spiny spider crab Maja brachydactyla in the northeastern Atlantic. Mar Ecol Prog Ser 362: 211–223.

9. Johnstone DL, O'Connell MF, Palstra FP, Ruzzante DE (2013) Mature male parr contribution to the effective size of an anadromous Atlantic salmon (*Salmo salar*) population over 30 years. Mol Ecol 22: 2394–2407.

10. Weir BS, Cockerham CC (1984) Estimating F-Statistics for the Analysis of Population-Structure. Evolution 38: 1358–1370.

11. Chapuis M-P, Estoup A (2007) Microsatellite null alleles and estimation of population differentiation. Mol Biol Evol 24: 621–631.

12. Meirmans PG, Hedrick PW (2011) Assessing population structure: F(ST) and related measures. Mol Ecol Resour 11: 5–18.

13. Jost L (2008) GST and its relatives do not measure differentiation. Mol Ecol 17: 4015–4026.

14. Cavalli-Sforza LL, Edwards AWF (1967) Phylogenetic analysis. Models and estimation procedures. Am J Hum Genet 19: 233.

15. Goudet J, Raymond M, de Meeus T, Rousset F (1996) Testing differentiation in diploid populations. Genetics 144: 1933–1940.

16. Lalis A, Leblois R, Liefried S, Ouarour A, Beeravolu CR, Michaux J, et al. (2016) New molecular data favour an anthropogenic introduction of the wood mouse (Apodemus sylvaticus) in North Africa. J Zool Syst Evol Res 54: 1-12.
